# The rationale and study design of two phase II trials examining the effects of BI 685509, a soluble guanylyl cyclase activator, on clinically significant portal hypertension in patients with compensated cirrhosis

**DOI:** 10.1186/s13063-023-07291-3

**Published:** 2023-04-24

**Authors:** Thomas Reiberger, Annalisa Berzigotti, Jonel Trebicka, Judith Ertle, Isabella Gashaw, Ros Swallow, Andrea Tomisser

**Affiliations:** 1 Division of Gastroenterology and Hepatology, Department of Internal Medicine III, Medical University of Vienna, Spitalgasse 23, 1090 Vienna, Austria; 2 Vienna Hepatic Hemodynamic Laboratory, Medical University of Vienna, Vienna, Austria; 3 Christian-Doppler Laboratory for Portal Hypertension and Liver Fibrosis, Medical University of Vienna, Vienna, Austria; 4 Department of Visceral Surgery and Medicine, Inselspital, Bern University Hospital, University of Bern, Bern, Switzerland; 5 Department of Internal Medicine B, University of Münster, Münster, Germany; 6 European Foundation for the Study of Chronic Liver Failure, EFCLIF, Barcelona, Spain; 7 Boehringer Ingelheim International GmbH, Ingelheim Am Rhein, Germany; 8 Boehringer Ingelheim Pharma GmbH & Co. KG, Ingelheim Am Rhein, Germany; 9 Boehringer Ingelheim Ltd, Bracknell, UK; 10 Boehringer Ingelheim RCV GmbH & Co. KG, Vienna, Austria

**Keywords:** Portal hypertension, Cirrhosis, Alcohol-related liver disease, Hepatitis B virus, Hepatitis C virus, Non-alcoholic steatohepatitis

## Abstract

**Background:**

Clinically significant portal hypertension (CSPH) drives cirrhosis-related complications (i.e. hepatic decompensation). Impaired nitric oxide (NO) bioavailability promotes sinusoidal vasoconstriction, which is the initial pathomechanism of CSPH development. Activation of soluble guanylyl cyclase (sGC), a key downstream effector of NO, facilitates sinusoidal vasodilation, which in turn may improve CSPH. Two phase II studies are being conducted to assess the efficacy of the NO-independent sGC activator BI 685509 in patients with CSPH due to various cirrhosis aetiologies.

**Methods:**

The 1366.0021 trial (NCT05161481) is a randomised, placebo-controlled, exploratory study that will assess BI 685509 (moderate or high dose) for 24 weeks in patients with CSPH due to alcohol-related liver disease. The 1366.0029 trial (NCT05282121) is a randomised, open-label, parallel-group, exploratory study that will assess BI 685509 (high dose) alone in patients with hepatitis B or C virus infection or non-alcoholic steatohepatitis (NASH) and in combination with 10 mg empagliflozin in patients with NASH and type 2 diabetes mellitus for 8 weeks. The 1366.0021 trial will enrol 105 patients, and the 1366.0029 trial will enrol 80 patients. In both studies, the primary endpoint is the change from baseline in hepatic venous pressure gradient (HVPG) until the end of treatment (24 or 8 weeks, respectively). Secondary endpoints include the proportion of patients with an HVPG reduction of >10% from baseline, the development of decompensation events and the change from baseline in HVPG after 8 weeks in the 1366.0021 trial. In addition, the trials will assess changes in liver and spleen stiffness by transient elastography, changes in hepatic and renal function and the tolerability of BI 685509.

**Discussion:**

These trials will enable assessment of the short-term (8 weeks) and longer-term (24 weeks) safety of BI 685509, and the effect of sGC activation by BI 685509 on CSPH due to various cirrhosis aetiologies. The trials will use central readings of the diagnostic gold standard HVPG for the primary endpoint, and changes in established non-invasive biomarkers, such as liver and spleen stiffness. Ultimately, these trials will provide key information for developing future phase III trials.

**Trial registration:**

1366.0021: EudraCT no. 2021–001285-38; ClinicalTrials.gov NCT05161481. Registered on 17 December 2021, https://www.clinicaltrials.gov/ct2/show/NCT05161481.

1366.0029: EudraCT no. 2021–005171-40; ClinicalTrials.gov NCT05282121. Registered on 16 March 2022, https://www.clinicaltrials.gov/ct2/show/NCT05282121.

## Administrative information

Note: the numbers in curly brackets in this protocol refer to SPIRIT checklist item numbers. The order of the items has been modified to group similar items (see http://www.equator-network.org/reporting-guidelines/spirit-2013-statement-defining-standard-protocol-items-for-clinical-trials/).

**Table:** 

Title {1}	The rationale and study design of two Phase II trials examining the effects of BI 685509, a soluble guanylyl cyclase activator, on clinically significant portal hypertension in patients with compensated cirrhosis
Trial registration {2a} {2b}	1366.0021 EudraCT No. 2021–001,285-38; ClinicalTrials NCT no. NCT05161481 1366.0029 EudraCT No. 2021–005,171-40; ClinicalTrials NCT no. NCT05282121
Protocol version {3}	1366.0021: c34798591-06 (version 6.0, 14/12/2022) 1366.0029: c36380139-04 (version 4.0, 15/12/2022)
Funding {4}	These studies are funded by Boehringer Ingelheim.
Author details {5a}	TR: 1 Division of Gastroenterology and Hepatology, Department of Internal Medicine III, Medical University of Vienna, Vienna, Austria 2 Vienna Hepatic Hemodynamic Laboratory, Medical University of Vienna, Vienna, Austria 3 Christian-Doppler Laboratory for Portal Hypertension and Liver Fibrosis, Medical University of Vienna, Vienna, Austria AB: Department for Visceral Surgery and Medicine, Inselspital, Bern University Hospital, University of Bern, Bern, Switzerland JT: 1 Department of Internal Medicine B, University of Münster, Münster, Germany 2 European Foundation for the Study of Chronic Liver Failure, EFCLIF, Barcelona, Spain JE: Boehringer Ingelheim International GmbH, Ingelheim am Rhein, Germany IG: Boehringer Ingelheim Pharma GmbH & Co. KG, Ingelheim am Rhein, Germany RS: Boehringer Ingelheim Ltd, Bracknell, UK AT: Boehringer Ingelheim RCV GmbH & Co. KG, Vienna, Austria
Name and contact information for the trial sponsor {5b}	1366.0021 Ros Swallow Boehringer Ingelheim Ltd Bracknell, Berkshire, UK Phone: + 44 1344 742535 Fax: + 44 1344 585479 1366.0029 Andrea Tomisser Boehringer Ingelheim RCV GmbH & Co. KG Dr. Boehringer-Gasse 5–11, A-1121 Wien, Austria Phone: + 43 1 80 105–8286 Fax: + 43 1 80 105–2375
Role of sponsor {5c}	Boehringer Ingelheim was involved in the design of the studies, the collection, analysis and interpretation of data, and writing the manuscript.

## Introduction

### Background and rationale {6a}

Liver cirrhosis (i.e. advanced chronic liver disease [ACLD]) develops as a consequence of various chronic liver diseases, with alcohol-related liver disease, hepatitis B virus (HBV), hepatitis C virus (HCV) and non-alcoholic steatohepatitis (NASH) representing the most common causes [1]. Importantly, although the relative contribution of HCV to worldwide liver-related mortality is constantly decreasing owing to direct antiviral agents that cure HCV infection in almost all patients, the obesity pandemic has resulted in a marked increase in NASH-related cirrhosis and death since 2005 [2]. Furthermore, alcohol-related liver cirrhosis represents a major cause of hospitalisation in Europe [2]. Cirrhosis is classified as compensated or decompensated, based on the presence of clinically evident complications (i.e. decompensating events, such as ascites and variceal haemorrhage) [3], which are associated with a considerably increased risk of mortality [4].

Hepatic venous pressure gradient (HVPG) is the current diagnostic gold standard for measuring portal pressure in ACLD [5] and has been shown to be more accurate than liver biopsy in predicting complications [3, 6]. An HVPG measurement of ≥ 10 mmHg defines clinically significant portal hypertension (CSPH) that identifies patients with compensated cirrhosis (compensated advanced chronic liver disease; cACLD) at particular risk for decompensation and mortality [7–9].

Therefore, the main therapeutic goal in patients with cACLD and CSPH is to prevent decompensation, which may be achieved by eliminating the primary aetiological factor of cirrhosis [3, 10, 11] and also by direct, effective treatment of CSPH [12, 13].

In many countries, non-selective beta-blockers (NSBBs) and carvedilol are used off-label as medical treatments for portal hypertension; they act by reducing portal venous inflow via a reduction in cardiac output (β_1_-adrenergic receptor blockade) and splanchnic blood flow (β_2_-adrenergic receptor blockade) [7, 10, 14]. However, contraindication against and intolerance to NSBBs limit their use in approximately 20–25% of patients [3, 15, 16]. In addition, not all patients achieve a haemodynamic response to NSBBs, and these patients show an insufficient decrease in HVPG [13].

Currently, there are no approved therapies that directly target or modulate the dynamic component of increased intrahepatic resistance (sinusoidal vasoconstriction), which is an early pathophysiological mechanism of portal hypertension and is largely due to an insufficient bioavailability of nitric oxide (NO) in the intrahepatic circulation [17]. Several therapies have been developed that address the imbalance of NO in patients with cirrhosis. Traditionally, isosorbide mononitrate has been used as an NO donor to treat portal hypertension [18]; however, systemic and renal adverse events (AEs) and a lack of efficacy have led to withdrawal of its recommendation from guidelines [19–21]. Several studies have demonstrated (with varying degrees of success) that statins can decrease portal pressure by ameliorating hepatic resistance and improving hepatic endothelial function in patients with cirrhosis, among other mechanisms, by increasing NO production [22–26].

Soluble guanylyl cyclase (sGC) is activated by NO to catalyse the formation of cyclic guanosine monophosphate [27, 28], a vasodilator and smooth-muscle relaxer [29]. The sGC pathway is disrupted in cirrhosis, leading to portal hypertension [30–32]. sGC modulators are a recently investigated treatment class with a broad range of therapeutic targets, including pulmonary hypertension, heart failure, hepatic fibrosis and portal pressure [29, 33–35]. sGC modulators can be classified into two types: sGC stimulators bind the reduced form of sGC and can act synergistically with NO, and sGC activators bind the oxidised, haem-free form of sGC, acting independently of NO in conditions of oxidative stress, such as cirrhosis [36, 37]. BI 685509 is an NO-independent sGC activator that reduces portal pressure, fibrosis and extrahepatic complications of portal hypertension in preclinical models of cirrhosis (data on file) and is currently being investigated in patients with portal hypertension to reduce portal pressure and to slow cirrhosis progression. In a phase Ib study in patients with mild and moderate hepatic impairment, multiple rising doses (MRDs) of BI 685509 were generally well tolerated and were associated with early signs of improved hepatic function [38]. Here, we discuss the rationale and design of two phase II, proof-of-concept trials of BI 685509 in patients with CSPH in compensated cirrhosis due to different aetiologies of cirrhosis.

### Objectives {1}

The 1366.0021 trial is a randomised, double-blind, placebo-controlled, exploratory trial assessing the effects of two different doses of BI 685509 on portal hypertension after 24 weeks of treatment in patients with CSPH in alcohol-related liver disease. The 1366.0029 trial is a randomised, open-label, parallel-group, exploratory trial assessing the effects of BI 685509 on portal hypertension after 8 weeks of treatment in patients with CSPH due to HCV infection, HBV infection or NASH and of the combination of BI 685509 and empagliflozin in patients with CSPH due to NASH and with type 2 diabetes mellitus (T2DM).

### Trial design {8}

Both trials will be conducted in compliance with the approved protocols and the ethical principles laid down in the Declaration of Helsinki and in accordance with the International Conference on Harmonization Good Clinical Practice Guideline, relevant Boehringer Ingelheim (BI) standard operating procedures, EU regulation 536/2014 and local regulations.

**Fig. 1:**
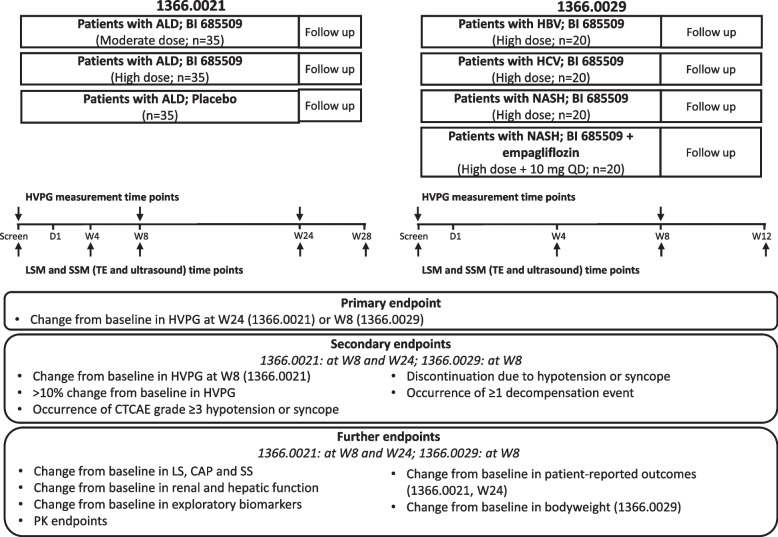
Designs of the 1366.0021 and 1366.0029 phase II trials *AE* adverse event, *ALD* alcohol-related liver disease, *BID* twice daily, *CAP* controlled attenuation parameter, *CTCAE* Common Terminology Criteria for Adverse Events, *D* Day, *HBV* hepatitis B virus, *HCV* hepatitis C virus, *HVPG* hepatic venous pressure gradient, *LS* liver stiffness, *LSM* liver stiffness measurement, *NASH* non-alcoholic steatohepatitis, *PK* pharmacokinetic, *QD* once daily, *SS* spleen stiffness, *SSM* spleen stiffness measurement, *TE* transient elastography, *W* week.

The 1366.0021 trial is a randomised, placebo-controlled and double-blind exploratory trial (Fig. 1). Two active dosing cohorts are planned: moderate- and high-dose oral BI 685509 twice daily (BID). A total of 105 patients will be enrolled, 35 to each active dose group and placebo. The 1366.0029 trial is a randomised and open-label exploratory trial (Fig. 1). Two dosing cohorts are planned: high-dose oral BI 685509 BID alone and high-dose oral BI 685509 BID with 10 mg oral empagliflozin once daily (QD). In total, 80 subjects will be enrolled; 20 patients each with HBV infection, HCV infection or NASH to the BI 685509 monotherapy cohort, and 20 patients with NASH with T2DM to the BI 685509 plus empagliflozin cohort.

## Methods: participants, interventions and outcomes

### Study setting {9}

The 1366.0021 trial will be conducted at approximately 73 centres across 23 countries worldwide, with approximately two patients randomised at each site. The 1366.0029 trial will be conducted at approximately 44 centres across 17 countries; all of these sites will also participate in the 1366.0021 trial.

### Eligibility criteria {10}

Adult patients with CSPH in compensated cirrhosis, defined by endoscopic proof of gastro-oesophageal varices or preventative endoscopic treatment of oesophageal varices and subsequent HVPG ≥ 10 mmHg, will be included in both studies.

In the 1366.0021 trial, eligible patients comprise those with alcohol-related compensated cirrhosis (based on histology or clinical evidence of cirrhosis), aged 18–75 years, who have abstained from significant alcohol misuse/abuse for ≥ 2 months prior to screening and are able to abstain from alcohol throughout the trial (based on investigator judgement; interviews on alcohol consumption will be conducted, and patients will be repeatedly advised to maintain abstinence). If receiving treatment with NSBBs or carvedilol, patients must be on a stable dose for ≥ 1 month prior to screening, with no planned dose change. If receiving treatment with statins, patients must be on a stable dose for ≥ 3 months prior to screening, with no planned dose change. Exclusion criteria include a previous clinically significant decompensation event (ascites extending beyond the perihepatic space, variceal bleeding, encephalopathy), systolic blood pressure of <100 mmHg, diastolic blood pressure of <70 mmHg, Model for End-Stage Liver Disease (MELD) score of >15, a Child–Pugh score of ≥ B8, an alanine or aspartate aminotransferase level of >5 × the upper limit of normal, an estimated glomerular filtration rate (eGFR) of <20 mL/min/1.73 m^2^ and an α-fetoprotein concentration of >50 ng/mL. Patients will also be excluded if they have an active SARS-CoV-2 infection, have had a previous orthotopic liver transplantation, have a planned or existing transjugular intrahepatic portosystemic shunt, have known portal vein thrombosis or have a history of clinically relevant orthostatic hypotension or other aetiologies of chronic liver disease. Patients with comorbid or prior HCV infection must not have received curative antiviral therapy within the last 2 years prior to study inclusion or, if they received curative antiviral therapy >2 years ago, must show sustained virological response at screening.

In the 1366.0029 trial, eligible patients will comprise those with compensated cirrhosis due to NASH with or without T2DM, HCV infection or HBV infection, based on histology or clinical evidence of cirrhosis. Eligibility criteria, additional or different to the 1366.0021 trial, include the following: patients with HCV must have achieved sustained virological response for ≥ 2 years prior to study inclusion and have no detectable HCV RNA, and patients with HBV infection receiving antiviral therapy must be on a stable dose for ≥ 6 months prior to screening, with no planned dose change and no detectable HBV DNA. Patients will be excluded if they are not receiving adequate treatment for NASH, HBV infection or HCV infection.

### Who will take informed consent? {26a}

Receipt of a signed and dated informed consent form is required for inclusion in the trial. A trial investigator or delegate will obtain informed, freely given written consent from each patient via a consent form, after confirming that the patient understands the content of the form. The investigator or delegate will then sign and date the consent form. If a trial collaborator has given a supplementary explanation to the patient, the trial collaborator will also sign and date the consent form.

### Additional consent provisions for collection and use of participant data and biological specimens {26b}

On the consent form, participants will be informed that should they choose to withdraw from the trial, the data collected up until that point will still be used to ensure correct completion and documentation of the trial. Any samples collected during the trial that have not been analysed at the time of withdrawal will be destroyed. Participants will also be informed that the research team will share relevant data with individuals from the centres taking part in the research or from regulatory authorities, if relevant. Both trials will offer voluntary biobanking, and participants will be required to give a separate biobanking informed consent in accordance with local ethical and regulatory requirements.

### Interventions

#### Explanation for the choice of comparators {6b}

In the 1366.0021 trial, placebo will be used as a comparator and will be run on top of the current standard of care (i.e. concomitant stable doses of NSBBs or carvedilol, or previous variceal ligation). The 1366.0029 trial is open label, and there will be no direct comparator; it is a proof-of-concept study in non-cholestatic liver disease aetiologies other than alcohol-related cirrhosis. Results of this trial will be compared with 8-week data from the 1366.0021 trial.

#### Intervention description {11a}

The 1366.0021 trial will be a randomised, double-blind, placebo-controlled study. Dose selection was based on the safety and pharmacokinetic (PK) results from the completed phase I MRD hepatic impairment trial (1366.0020) [38]. The planned dosing cohorts are moderate- and high-dose oral BI 685509 (film-coated tablet) and matching placebo (film-coated tablet); the high dose is the maximum tolerated single dose observed in phase I trials. Due to ethical reasons, anticipated sub-therapeutic doses will not be included, as measurable differences are not expected between a very low dose and placebo. Trial medication will be dispensed at investigational sites, and patients will be given the appropriate number of medication kits [1–4].

All patients will start on a low dose of BI 685509 or matching placebo, which will be up-titrated to moderate-dose BI 685509 or matching placebo (pseudo-titration) if tolerated after 1 week (day 8), then further up-titrated to high-dose BI 685509 or matching placebo (pseudo-titration) at day 15 if tolerated (Table 1). Patients randomised to the moderate dose cohort will have a pseudo-titration at day 15, remaining on their current dose. Each patient will receive two film-coated oral tablets of BI 685509 or matching placebo for 24 weeks, with a 4-week follow-up period without trial medication.

**Table 1:**
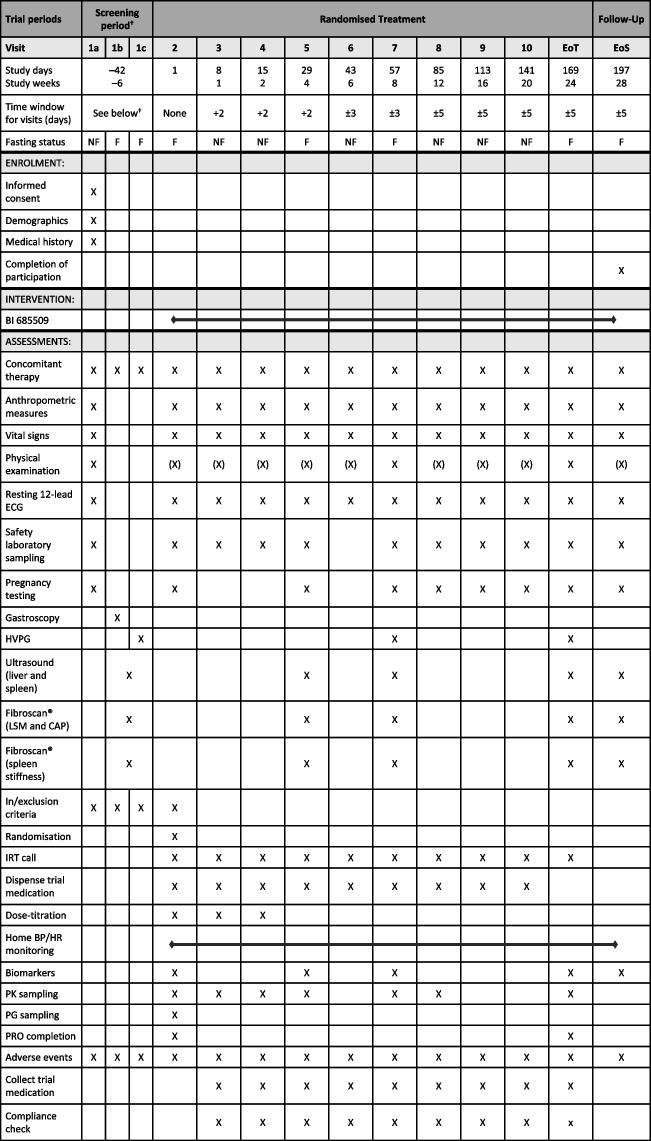
Endpoint measures and timings for the 1366.0021 trial

^†^Screening visits should be completed within 4 weeks; a maximum of 6 weeks (42 days) will be permitted

*BP* blood pressure, *CAP* controlled attenuation parameter, *ECG* electrocardiogram, *EoS* end of study, *EoT* end of treatment, *HR* heart rate, *HVPG* hepatic venous pressure gradient, *IRT* Interactive Response Technology, *LSM* liver stiffness measure, *PG* pressure gradient, *PK* pharmacokinetic, *PRO* patient-reported outcome

The 1366.0029 trial is an open-label study with active treatment only. As this study is shorter in duration than the 1366.0021 trial (8 weeks vs 24 weeks), all patients will receive a high BI 685509 (film-coated tablet) maintenance dose. The dose that will be used in this study is predicted to achieve pharmacologically relevant exposure in patients with hepatic impairment, and is the maximum tolerated single dose, as identified in the phase I 1366.0020 trial [38]. Trial medication will be dispensed at investigational sites, and patients will be given the appropriate number of medication kits (one or two). All patients will follow the same up-titration scheme as the high-dose BI 685509 cohort from the 1366.0021 trial (Table 2). Each patient will receive one film-coated oral tablet of BI 685509 for 8 weeks, with a 4-week follow-up period without trial medication. Patients in the empagliflozin arm will receive an additional film-coated oral empagliflozin tablet (10 mg QD) for 8 weeks.

**Table 2:**
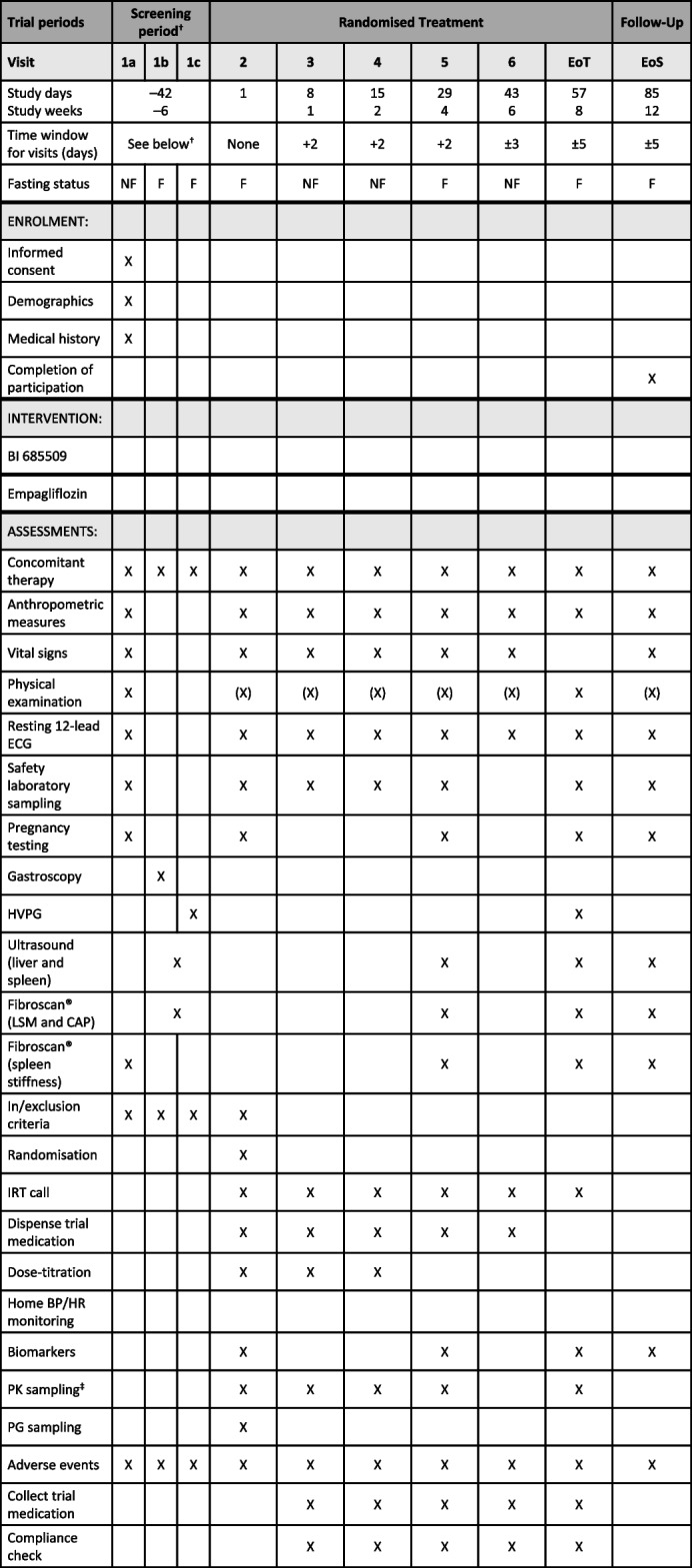
Endpoint measures and timings for the 1366.0029 trial

^†^Screening visits should be completed within 4 weeks; a maximum of 6 weeks (42 days) will be permitted

^‡^PK sampling of BI 685509 and empagliflozin

*BP* blood pressure, *CAP* controlled attenuation parameter, *ECG* electrocardiogram, *EoS* end of study, *EoT* end of treatment, *HR* heart rate, *HVPG* hepatic venous pressure gradient, *IRT* Interactive Response Technology, *LSM* liver stiffness measure, *PG* pressure gradient, *PK* pharmacokinetic, *PRO* patient-reported outcome

In each trial, it is recommended that BI 685509 doses are taken in the morning and evening, at approximately the same time every day, with ≥ 10 h between each dose. Empagliflozin doses should be taken with the first daily dose of BI 685509.

#### Criteria for discontinuing or modifying allocated interventions {11b}

Patients may discontinue treatment or withdraw consent to participate in either trial at any time without any justification. They may also discontinue if they are repeatedly non-compliant with important trial procedures, need to take concomitant medication that interferes with the safety or efficacy of the trial treatment, experience a severe infection, meet the criteria for hepatic injury (increased liver function tests), have an acute liver decompensation event, have worsening of liver function, have a QT/QTcF interval of >500 ms or an increase of QT/QTcF of >60 ms from baseline, have not successfully completed the dose-titration period or become pregnant. If a patient permanently discontinues trial medication, an early discontinuation visit is required within 7 days of medication discontinuation, and a follow-up visit should be performed 4 weeks after the early discontinuation visit. The investigator may discontinue patients at any time, based on their clinical judgement.

If a patient has an AE that may be related to BI 685509 treatment, based on the investigator’s judgement, trial medication can be interrupted, or the dose can be reduced if the patient has successfully completed dose titration.

#### Strategies to improve adherence to interventions {11c}

Patients will be assessed regularly at scheduled visits (Tables 1 and 2), with more frequent visits during the dose-titration phases of the trials. Patients will also be instructed to bring all unused trial medication and empty packaging to the scheduled visits.

#### Relevant concomitant care permitted or prohibited during the trials {11d}

Stable doses of concomitant therapies for chronic conditions are permissible throughout the trials, provided that neither the condition nor treatment leads to exclusion of the patient. Specifically, NSBB or carvedilol use is permitted, but these concomitant therapies should not be newly initiated during the trials; endoscopic variceal ligation is permitted during the trials, as required. Patients who develop oedema or gastrointestinal events will be managed by standard of care, such as diuretics. Use of NO–sGC–cyclic guanosine monophosphate pathway-activating therapies (NO donors, phosphodiesterase-5 inhibitors, non-specific phosphodiesterase inhibitors or sGC stimulators), organic anion-transporting polypeptide 1B1/3 inhibitors, concomitant therapies known to inhibit or induce uridine glucuronyl transferase enzymes or concomitant therapies with a known risk of torsade de pointes are not allowed during participation in the trials. In the 1366.0029 trial, the use of other sodium glucose co-transporter 2 or sodium glucose co-transporter 1/2 inhibitors is not allowed in patients with NASH. These restrictions apply from within five half-lives prior to randomisation until the end of the study; therapies with a known risk of torsade de pointes are restricted from screening until the end of the study. If patients are taking concomitant therapy that is metabolised by cytochrome P450 (CYP) 3A4 and/or CYP2C8 which has a narrow therapeutic index and/or is a sensitive substrate, close monitoring for AEs is recommended.

#### Provisions for post-trial care {30}

Patients will receive continued care according to the standard operating procedures of each centre after the end of the study.

### Outcomes {12}

The primary endpoint for each trial is the percentage change in HVPG from baseline after 24 weeks (1366.0021) or 8 weeks (1366.0029) of treatment. Secondary endpoints are the percentage change in HVPG from baseline after 8 weeks (1366.0021 trial only); response defined as a >10% reduction in HVPG from baseline; occurrence of Common Terminology Criteria for Adverse Events grade ≥ 3 hypotension or syncope or discontinuation due to hypotension or syncope; and the occurrence of one or more decompensation events. Each secondary endpoint will be assessed after 8 weeks in both trials and after 24 weeks in the 1366.0021 trial.

Further endpoints in both trials include (but are not limited to) change from baseline in spleen stiffness, liver fat content and liver stiffness by transient elastography, hepatic function (measured by prothrombin time/international normalised ratio, activated partial prothrombin time, bilirubin, liver enzymes [alanine aminotransferase, aspartate aminotransferase, γ-glutamyl transferase and alkaline phosphatase] and albumin), renal function (measured by eGFR) and disease-specific and mode-of-action exploratory biomarkers.

In the 1366.0021 trial, the change from baseline in further endpoints will be assessed at the main time points (8 and 24 weeks) and will be measured at additional time points as defined in Table 1. This study will also assess disease-specific and generic patient-reported outcomes (PROs) until 24 weeks of treatment.

In the 1366.0029 trial, all further endpoints will be assessed after 8 weeks of treatment. This trial will also assess the change from baseline in metabolic function (measured by HbA1c, serum glucose and Homeostatic Model Assessment for Insulin Resistance), bodyweight and hepatic function (measured by systemic hepatic filtration rate and portal hepatic filtration rate).

Plasma concentrations of BI 685509 and, if feasible, further PK endpoints will be determined in both trials: maximum concentration (C_max_), time to maximum concentration (t_max_), area under the concentration–time curve over time period t1–t2 after single dose administration (AUC_t1–t2_) and pre-dose concentration (C_pre_) will be measured following N doses and at steady state. In the empagliflozin combination arm of the 1366.0029 trial, all PK endpoints for BI 685509 will also be determined for empagliflozin, if feasible.

### Participant timeline {13}

The participant timeline is shown in Tables 1 and 2.

### Sample size {14}

The 1366.0021 trial will enrol 105 patients, randomised 1:1:1 to each dose group. Sample size was calculated based on the probability of observing the estimated treatment effect for mean reduction in HVPG from baseline. It was assumed that the mean reduction in HVPG from baseline at week 24 would be 0%, 23% and 25% for the placebo, moderate and high dose groups, respectively. With a sample size of 35 patients per dose group, there is an 84.8% probability of observing a difference of ≥ 20% reduction in HVPG from baseline between at least one BI 685509 dose group and placebo. An interim analysis will take place after 60 patients (20 per dose group) have completed the first 8 weeks of treatment and the week 8 HVPG measurement. With this sample size, the probability of observing a treatment effect is 87.7%, assuming that the mean reduction in HVPG from baseline will be 0%, 17% and 20% for the placebo, moderate and high dose groups, respectively.

The 1366.0029 trial will enrol 80 patients: 20 patients each with HBV infection, HCV infection or NASH receiving BI 685509 monotherapy and 20 patients with NASH and T2DM receiving BI 685509 and empagliflozin. Sample size was calculated based on the probability of observing the estimated treatment effect for mean reduction in HVPG from baseline. With a sample size of 20 patients per treatment group, there is a 96.3% probability of observing a ≥ 10% mean reduction in HVPG from baseline.

### Recruitment {15}

Materials for patients have been developed to inform and support the recruitment of patients identified at participating centres. In addition, HVPG educational materials for patients, including a video animation of the procedure, have been created to support patient recruitment and retention. Patients will be recruited at academic centres focused on the care of patients with cirrhosis and that have established facilities for HVPG measurements. Importantly, referral centres should refer appropriate patients with compensated cirrhosis of non-cholestatic aetiology and known presence of varices and/or a history of variceal band ligation to study sites for screening.

## Assignment of interventions: allocation

### Sequence generation {16a}

In the 1366.0021 trial, patients will be randomised 1:1:1 via a validated system (Interactive Response Technology [IRT]) to receive placebo, moderate or high dose BI 685509, stratified by the use of NSBBs or carvedilol. In the 1366.0029 trial, patients will be assigned to their treatment group using IRT based on underlying disease aetiology (HBV, HCV or NASH), and all patients will receive high dose BI 685509. In addition, a subgroup of patients with NASH and T2DM will be randomised 1:1 via IRT to receive BI 685509 alone or in combination with empagliflozin. The randomisation list for each group will be generated using IRT that uses a pseudorandom number generator so that the assignment of treatment will be reproducible and non-predictable.

### Concealment mechanism {16b}

All patients screened in each study must be registered with IRT. In the 1366.0021 trial, patients will be randomised at visit 2 to receive either active treatment or placebo according to a randomisation plan using IRT. IRT will be used to conceal the sequence until intervention assignment.

### Implementation {16c}

In the 1366.0021 trial, each centre will use IRT to randomise patients. The allocation sequence will be based on a randomisation list generated by the trial sponsor, and treatment assignment will be controlled by the IRT system.

## Assignment of interventions: blinding

### Who will be blinded {17a}

The 1366.0021 is a double-blind trial, with all patients, investigators, central reviewers and all other individuals involved in trial conduct or analysis remaining blinded until the database is declared ready for analysis. Randomisation codes will be provided to bioanalysts to allow for the exclusion of the placebo group from the PK analyses. The randomisation codes and results of the analyses will not be disclosed until after the database lock and will be provided to the bioanalysts prior to the trial completion of the last patient to allow the exclusion of patients receiving placebo from the PK analysis. A data monitoring committee will perform unblinded safety evaluations throughout the trial to ensure that patients are protected from potential harm. An independent statistician will prepare tables and listings from all information provided to the committee in an unblinded fashion. The 1366.0029 trial will be open-label and unblinded, with all patients receiving BI 685509 alone or in combination with empagliflozin. For both studies, an independent data monitoring committee will perform an unblinded safety evaluation at specific intervals.

### Procedure for unblinding if needed {17b}

Emergency unblinding in the 1366.0021 trial will be available to the investigator via IRT and will be used only in an emergency when the identity of the trial drug must be known by the investigator to provide appropriate medical treatment or otherwise ensure the safety of trial participants.

## Data collection and management

### Plans for assessment and collection of outcomes {18a}

The endpoint assessments will be identical between the trials until week 8 (visit 7 in 1366.0021, end of treatment in 1366.0029; Tables 1 and 2). For all efficacy and safety endpoints, the baseline will be defined as the last observed measurement prior to the administration of the trial medication. All onsite assessments will be performed by a qualified person.

The change from baseline in HVPG will be assessed at the end of treatment in both trials and at visit 7 in the 1366.0021 trial. HVPG measurements will be conducted in a standardised fashion at all sites, with wedged hepatic venous pressure and free hepatic venous pressure measured in triplicate, using the same hepatic vein, prior to administration of trial medication and after an overnight fast. All HVPG measurements will be performed at approximately the same time of day as the baseline measurement. At baseline, the HVPG measurement will be assessed locally for enrolment. Measurements within the trial will be assessed by a central reader blinded to the time point of the trace. The central reader will also assess the results of the baseline HVPG measurement and will be favoured over the local interpretation in the case of any discrepancies.

Safety will be assessed in both trials in terms of AEs, physical examination, vital signs and home blood pressure and heart rate monitoring, safety laboratory parameters, 12-lead electrocardiogram (ECG), ultrasound (liver and spleen) and gastroscopy (baseline only). Physical examination will be performed at visit 1 and the end of treatment in both trials and at visit 7 in the 1366.0021 trial, with further examinations performed only if required. Vital signs measured at trial site visits will be evaluated prior to blood sampling and 12-lead ECG measurements. From visit 2 onwards, patients will use home blood pressure monitoring equipment. ECGs will be performed after the assessment of vital signs and prior to blood sampling and administration of trial medication. During the dose-titration period, 12-lead ECGs and vital sign assessments will also be performed approximately 1 and 2 h after dosing. Blood samples for safety laboratory parameters should be drawn prior to the administration of trial medication and the fasting status recorded. The central laboratory will provide laboratory reports to the investigator, and clinically relevant findings, as judged by the investigator, will be reported as AEs. Ultrasound imaging of the liver and spleen will be performed after an overnight fast. A gastroscopy will be performed at screening after an overnight fast; a patient may miss this gastroscopy if they have had this procedure within 6 months prior to screening, with documentary evidence confirming the presence of oesophageal/gastric varices.

Change in spleen stiffness, liver stiffness and liver fat content from baseline will be assessed using a FibroScan® Expert 630 at visit 5, the end of treatment and follow-up in both trials and at visit 7 in the 1366.0021 trial, ideally on the same day as ultrasound examinations. Measurements will be taken after an overnight fast. Liver and spleen stiffness will be assessed by vibration-controlled transient elastography, and liver fat content will be assessed using a controlled attenuation parameter.

Exploratory biomarker assessments (including safety and liver function and injury) will take place at baseline, visit 5, the end of treatment and follow-up in both trials and at visit 7 in the 1366.0021 trial (Table 3). Trial samples will be discarded after the completion of the additional investigations and no later than 5 years after the final report has been signed. Biomarkers will be evaluated for the treatment monitoring (change from baseline) and for potential to predict treatment response (correlation of baseline data with treatment outcomes). All biomarker samples will be collected after an overnight fast.

**Table 3:** Safety and key exploratory biomarkers

Parameter	Specific measure
Safety laboratory measurements of liver function	PT/INR aPTT Bilirubin (direct and indirect) Liver enzymes (ALT, AST, γGT and alkaline phosphatase) Albumin Ferritin
Composite scores of advanced liver disease	Fibrosis-4 index FibroScan®-AST score FibroScan®-based scores for advanced fibrosis (agile 3 +) and indicative of liver cirrhosis (agile 4)* MELD MELD-Na ELF score NIS4™ score
Cardiac and renal biomarkers	BNP* Troponin I eGFR Creatinine
Fibrosis markers	Hyaluronic acid Procollagen III amino-terminal peptide TIMP1 Pro-C3 Pro-C6*
Inflammation and cell death biomarkers	hs-CRP Alpha 2 macroglobulin CK18 M30 CK18 M65 CK19 fragment CYFRA21-1
Metabolic function biomarkers	Apolipoprotein A1 HbA1c* Serum glucose HOMA-IR*
Alcohol abuse biomarker^§^	Phosphatidylethanol
Bile acid panel	AbsoluteIDQ® bile acids kit (Biocrates) covering 20 bile acids
miRNA profiling	RNA sequencing from plasma
Pharmacogenetics	Known genetic variants such as rs738409 (in *PNPLA3*) and rs58542926 (in *TM6SF2*)

^*^Assessed in the 1366.0029 trial only

^§^Assessed in the 1366.0021 trial only

*γGT* gamma-glutamyl transferase, *ALT* alanine aminotransferase, *aPTT* activated partial thromboplastin time, *AST* aspartate aminotransferase, *BNP* brain natriuretic peptide, *CK18 M30* caspase-cleaved cytokeratin 18, *CK18 M65* total cytokeratin 18, *CK19* cytokeratin 19, *eGFR* estimated glomerular filtration rate, *ELF* enhanced liver fibrosis, *HOMA-IR* Homeostatic Model Assessment for Insulin Resistance, *hs-CRP* high-sensitivity C-reactive protein, *MELD* Model for End-Stage Liver Disease, *MELD-Na* Model for End-Stage Liver Disease-sodium, *Pro-C3* amino-terminal propeptide of type III collagen, *Pro-C6* propeptide of type VI collagen, *PT/INR* prothrombin time/international internalised ratio, *TIMP1* tissue inhibitor of metalloproteinase 1

PK sampling will take place at visits 2, 3, 4 and 5 and at the end of treatment in both trials and additionally at visits 7 and 8 in the 1366.0021 trial. For quantification of plasma concentrations of BI 685509, blood will be taken from an antecubital or forearm vein, and the date, time and fasting status of each patient will be recorded before the data and calculated PK parameters are tabulated and displayed graphically. In the 1366.0029 trial, PK parameters will be determined for both BI 685509 and empagliflozin in the combination arm.

In the 1366.0021 trial, PROs will be assessed at baseline and the end of treatment and will be completed with a consistent approach for each patient. Generic health-related quality of life will be assessed using the EQ-5D-5 Levels and the 36-Item Short Form Health Survey version 2, and disease-specific health-related quality of life will be assessed by the Chronic Liver Disease Questionnaire.

### Plans to promote participant retention and complete follow-up {18b}

The coordinating investigator of each trial will work with the trial sponsors and investigators to ensure subject enrolment and retention. Measures to minimise the patient withdrawal rate include careful patient selection, appropriate explanation of the trial requirements and procedures prior to first administration of trial medication and explanation of the differences between trial medication discontinuation and withdrawal of consent. If a patient has an AE that is judged by an investigator to be treatment-related, treatment can be interrupted or the dose can be reduced providing that the patient has completed the dose-escalation period.

### Data management {19}

Data management will be conducted in accordance with BI’s standard operating procedures and industry standards. BI’s data management process supports International Council for Harmonisation of Technical Requirements for Pharmaceuticals for Human Use Good Clinical Practice. Data will be collected directly via an electronic case report form or external data upload (e.g. for central laboratory results). In accordance with regulatory requirements, the centre trial investigator will prepare and maintain source documents and trial records for each patient, including all observations and other data pertinent to the investigation. Coding will be performed according to the current available dictionaries, with updates twice per year; range checks will be established during database set-up. The Medidata Rave Electronic Data Capture system will be used for database set-up and conduct. The investigator/institution will allow trial-related monitoring, audits, institutional review board (IRB)/independent ethics committee (IEC) review and regulatory inspections. Direct access will be provided to all source documents/data, including progress notes and copies of laboratory and medical test results, which must be available at all times for review by the clinical research associate, auditor and regulatory inspector.

All data management procedures are documented in the trial Data Management Plan (DMP), which describes the processes and procedures for the collection, processing and quality control of clinical trial data performed by Data Management throughout the lifecycle of the clinical trial; these processes and procedures are implemented to create and maintain high-quality data collection systems and to ensure data integrity. The DMP serves as a communication and reference tool for clinical trial teams to create and maintain a high-quality database for analysis.

### Confidentiality {27}

Data protection and data security measures will be implemented for the collection, storage and processing of patient data in accordance with principles 6 and 12 of the World Health Organization Good Clinical Research Practice handbook. Participants’ data will be collected via a secure electronic data capture system. System access rights will be controlled by BI, and dataset access will be limited to BI. BI endorses the Principles for Responsible Clinical Trial Data Sharing set out by the Pharmaceutical Research and Manufacturers of America and the European Federation of Pharmaceutical Industries and Associations. BI provides redacted clinical study reports and clinical documents on request. Furthermore, BI provides qualified scientific and medical researchers access to de-identified, analysable patient-level clinical study data. Further information can be found on BI’s Data Transparency website (https://www.mystudywindow.com/).

Individual patient data obtained as a result of this trial are considered confidential, and disclosure to third parties is prohibited with the following exception: personalised treatment data may be given to the patient’s personal physician or to other appropriate medical personnel responsible for the patient’s welfare. Data generated at the site as a result of the trial will be available for inspection on request by the participating physicians, the sponsor’s representatives, the IRB/IEC and regulatory authorities.

Before providing any copy of patient source documents to the sponsor or external suppliers, the investigator will ensure that all patient identifiers (e.g. name, initials, address, telephone number and social security number) have been removed or redacted.

### Plans for collection, laboratory evaluation and storage of biological specimens for genetic or molecular analysis in this trial/future use {33}

Plasma and serum for biobanking will be collected in a fasted state at visit 2 and the end of treatment in both trials and at visit 7 in the 1366.0021 trial. All BI internal and external facilities storing biological samples from patients will be qualified for the storage of clinical biological samples. An appropriate sample and data management system is in place, including an audit trail for clinical data and samples to identify and destroy such samples according to the informed consent. Detailed instructions on sampling, preparation, processing, shipment and storage will be provided to investigators in the central laboratory manual. There is no genetic element to the biobanking planned in these trials.

## Statistical methods

### Statistical methods for primary and secondary outcomes {20a}

No testing of statistical hypotheses is planned; descriptive statistics will be provided for each endpoint; therefore, both trials are exploratory in nature. In the 1366.0021 trial, the endpoints will be investigated in comparison to placebo; however, it is not planned to test a statistical hypothesis with regard to these variables in a confirmatory matter. The primary endpoint analysis will use a restricted maximum likelihood-based approach using a mixed model with repeated measurements to obtain adjusted means for the treatment effects. This will include the fixed categorical effects of treatment at each visit, the use of NSBBs or carvedilol at baseline and the continuous effects of baseline HVPG at each visit (Table 1). Visits will be treated as a repeated measure with an unstructured covariance structure to model the within-patient measurements. The Kenward–Roger approximation will be used to estimate the denominator degrees of freedom and to adjust standard errors. In the 1366.0029 trial, the primary endpoint will be analysed using an ANCOVA model, including ‘treatment’ as a fixed classification effect and ‘HVPG at baseline’ as a linear covariate. These analyses will be used for the estimation of treatment effects without performing statistical tests.

### Interim analyses {21b}

In the 1366.0021 trial, an interim analysis is planned after the first 60 patients (20 per dose group) have completed the first 8 weeks of treatment and the week 8 HVPG procedure. All available data, except data for the primary endpoint and one secondary endpoint (week 8 HVPG), will be analysed the same way as the final analysis. The percentage change in HVPG from baseline after 8 weeks of treatment will be analysed by ANCOVA. The interim analysis will be performed by an independent statistical and programming team; there will be no changes in trial design as a result of the interim analysis.

### Methods for additional analyses (e.g. subgroup analyses) {20b}

Correlation analyses of exploratory biomarkers and assessment of response to treatment with key endpoints will be performed.

### Methods in analysis to handle protocol non-adherence and any statistical methods to handle missing data {20c}

The handling of missing PK data in both trials will be performed according to the relevant BI internal procedures. In the 1366.0021 trial, the mixed effect model for the primary endpoint will handle missing data based on a likelihood method under the ‘missing at random’ assumption. No imputation of missing data is planned for the remaining endpoints of either trial or the interim analysis of week 8 HVPG data in the 1366.0021 trial. The decision to exclude patients from analysis sets due to protocol deviations will be taken during the course of the trials and finalised at the last report planning meeting.

### Plans to give full access to the full protocol, participant-level data and statistical code {31c}

The current trial protocols have been shared in this manuscript. No patient-level data will be shared to third parties while the trials are ongoing.

## Oversight and monitoring {21}

### Composition of the coordinating centre and trial steering committee {5d}

For these trials, there will be no steering committee or coordinating centre. However, the coordinating investigators will work with the sponsors and investigators to ensure subject enrolment and retention.

### Composition of the data monitoring committee, its role and reporting structure {21a}

A data monitoring committee has been established to periodically evaluate the available accrued trial data (including unblinded safety data, efficacy data, results of interim analyses for the 1366.0021 trial, final results of the 1366.0029 trial, significant safety concerns and decisions from hepatic injury adjudication) and to recommend to the sponsor the continuation, modification or termination of the trial. The data monitoring committee is independent from BI and includes a statistician and physicians experienced in the treatment of liver cirrhosis. Regular committee meetings will be held quarterly. Details of the data monitoring committee’s responsibilities and procedures are available in the prespecified data monitoring committee charter. The primary role of the data monitoring committee is the ongoing evaluation of safety.

### Adverse event reporting and harms {22}

The investigator must immediately (within 24 h) report to the sponsor’s unique entry point serious adverse events (SAEs), AEs of special interest and non-SAEs that are relevant to the reported SAE or AE of special interest, using the Boehringer Ingelheim SAE form. The same timeline will apply if follow-up information becomes available.

### Frequency and plans for auditing trial conduct {23}

Should an audit or inspection of either trial be conducted, the quality assurance auditor will have access to all medical records, the investigator’s trial-related files and correspondence, and the informed consent documentation.

### Plans for communicating important protocol amendments to relevant parties (e.g. trial participants, ethical committees) {25}

Important protocol amendments will be listed on the ClinicalTrials.gov webpage for each trial and be made available via the BlueSky training portal, clinical trial managers and the BI clinical trial portal Clinergize. Any deviations from the protocol will be fully documented. The principal investigator is responsible for IEC/IRB submissions according to local regulations.

### Dissemination plans {31a}

The results of these trials will be disseminated at relevant congresses when interim data become available and as a full formal publication after the last patient has finished treatment and after analyses have been completed. Plain language summaries of the study results will be developed by Boehringer Ingelheim and made freely available on the MyStudyWindow web centre (https://www.mystudywindow.com/).

### Informed consent materials

A sample informed consent document is provided as supplementary information. This document will be adapted in each country in line with the corresponding language, as well as national and local regulations.

## Discussion

Currently, there are no approved therapies for patients with CSPH in compensated cirrhosis that directly target the increase in intrahepatic vascular resistance as pathophysiological mechanisms of portal hypertension. BI 685509, an sGC activator, has the potential to modulate portal pressure; these studies aim to assess the effects of BI 685509 on portal hypertension in patients with CSPH in cirrhosis due to various aetiologies of liver disease. Between both trials, patients with the four most common aetiologies of liver cirrhosis are included (alcohol-related liver disease, HBV infection, HCV infection and NASH). In a previous phase I MRD trial of BI 685509 in patients with mild and moderate hepatic impairment, no differences in PK parameters of BI 685509 were seen between patients with alcohol-related liver disease, chronic viral hepatitis (HBV or HCV infection) and NASH [38]. Therefore, comparisons of PK parameters and pharmacodynamics between the two trials presented here after 8 weeks of treatment will be possible. Importantly, patients with HCV achieving sustained virological response following treatment with direct-acting antivirals represent an important population of patients who remain at risk of portal hypertension-related complications despite aetiological cure [39, 40]; no treatment is available in this setting to date.

HVPG is the current gold standard for assessing the severity of sinusoidal portal hypertension in patients with cirrhosis. The test–retest reliability of HVPG measurements has been shown to be high in the context of clinical studies, particularly those limited to patients with compensated cirrhosis, such as in the trials presented here [41]. Furthermore, most of the data demonstrating the association between a decrease in HVPG and a reduction in clinical outcomes are based on trials of NSBBs [42, 43]; therefore, drugs with different mechanisms of action for reducing portal hypertension will require validation as they may result in a different association between HVPG and clinical outcomes [14]. However, current guidelines still recommend the use of HVPG in clinical trials [10], with a decrease in baseline HVPG being recommended as the main endpoint in phase II trials [14], such as in the 1366.0021 and 1366.0029 studies. In these trials, response is defined as a >10% reduction in HVPG from baseline; this cut-off value is used to define clinically relevant responses to aetiological therapies and NSBBs [10, 44, 45]. In the 1366.0021 trial, patients will be stratified by the use of NSBBs or carvedilol as these therapies can influence the HVPG-lowering effect. In addition, patients with alcohol-related cirrhosis will be separated from patients with cirrhosis due to other aetiologies per regulatory request, because patients with alcohol-related cirrhosis have shown lower variability and lower absolute changes in HVPG [41].

Liver and spleen stiffness measurements via transient elastography are emerging non-invasive tests for assessing portal hypertension [46, 47]. Although HVPG measurements are the current gold standard, they are invasive, expensive and not commonly used in clinical practice [47]; liver and spleen stiffness measurements correlate well with HVPG and CSPH [48–51]. Liver and spleen stiffness measurements can be used to complement HVPG results and could lead to a reduction in the need for invasive measures of disease. Although HVPG remains the only validated tool for the exact assessment of the severity of portal hypertension, liver and spleen stiffness measurements can be used to rule in or out CSPH and cACLD and to refine the risk of high-risk varices [52]. Moreover, changes in spleen stiffness measurements have been shown to reflect changes in HVPG in patients receiving NSBB therapy [53, 54]. Therapies that are vasoactive in nature, such as BI 685509, can produce rapid and dynamic changes that may require repeated measurements, something that is difficult to achieve with invasive techniques such as HVPG. Therefore, guidelines recommend that research should focus on developing and validating non-invasive measures, such as liver and spleen stiffness, to predict decompensation and identify high-risk patients [10]. The previous phase Ib MRD study of BI 685509 was the first clinical study to assess spleen stiffness as a surrogate endpoint for portal hypertension [38]. Although the spleen stiffness results of this trial were inconclusive, the larger sample sizes of the 1366.0021 and 1366.0029 trials and the trial site qualification process implemented in the trial centres may help to overcome these issues. Disruption caused by the COVID-19 pandemic made training for the novel FibroScan® device difficult in the Phase Ib trial; however, more rigorous training and certification on the use of this device in the trial centres will improve repeatability [55]. The use of spleen stiffness measurements in these studies alongside HVPG can help in further investigating patients’ status and gathering insights into the use of this method to investigate portal hypertension.

The treatment duration of 8 weeks in the 1366.0029 trial and the interim analysis at week 8 in the 1366.0021 trial will allow for the evaluation of short-term efficacy data from HVPG measurements and other non-invasive measures and for indirect comparisons between the two trials. The longer-term (24 weeks) assessment in the 1366.0021 trial will also investigate the potential for the adaptation to sGC activation on portal pressure with chronic treatment and the assessment of the maintenance of the response to therapy. HVPG can be considered to be an objective assessment with standardised procedures. Conducting central reads of the HVPG measurements, and using the same central reader for both trials, enhances confidence and increases the reliability of data. Furthermore, both trials will be conducted at the same study centres, improving the comparison of trial data from different aetiologies, and the global nature of the trials allows for a broad coverage of patients. The standardisation of transient elastography evaluations by using the same device across sites, standardised training of site personnel and, if possible, the use of the same operator for the assessments for a single patient will produce comparable results between the two trials.

In the 1366.0021 trial, patients are required to abstain from significant alcohol misuse/abuse for at least 2 months prior to screening and abstain completely throughout the trial. For patients with an alcohol-related disease, this may be difficult and may present an increased risk of protocol deviation. To mitigate this, patient-reported information on abstinence from alcohol will be collected, and phosphatidylethanol, an alcohol-specific biomarker, will be measured during the treatment period. Furthermore, the maximum duration of treatment will be 24 weeks in the 1366.0021 trial, and hence no long-term data will be collected, and limited data will be obtained on whether sGC activation prevents other complications or decompensation. The 1366.0029 trial is not placebo-controlled and, with the exception of the NASH population, is not randomised. Although placebo data will be collected in the 1366.0021 trial, any comparisons with the other aetiologies will be indirect. Due to the modest patient numbers, these trials will not allow the collection of information on potential ethnic differences in treatment outcomes. However, based on the PK profile in phase I trials, no differences between major ethnicities are expected.

The results of the presented trials will inform the clinical development of BI 685509 in pivotal phase III trials, which are intended to examine clinical endpoints.

### Trial status

For the 1366.0021 trial, the first patient was screened on 27 April 2022, and the first patient for the 1366.0029 trial was screened on 28 June 2022. The trials will be completed by approximately 25 March 2024 and 20 November 2023, respectively.

## Data Availability

To ensure independent interpretation of clinical study results and enable authors to fulfil their role and obligations under the ICMJE criteria, Boehringer Ingelheim grants all external authors access to relevant clinical study data. In adherence with the Boehringer Ingelheim Policy on Transparency and Publication of Clinical Study Data, scientific and medical researchers can request access to clinical study data after publication of the primary manuscript and secondary analyses in peer-reviewed journals and regulatory and reimbursement activities are completed, normally within 1 year after the marketing application has been granted by major Regulatory Authorities. Researchers should use the https://vivli.org/ link to request access to study data and visit https://www.mystudywindow.com/msw/datasharing for further information.

## Abbreviations

γGT: Gamma-glutamyl transferase
ACLD: Advanced chronic liver disease
AE: Adverse event
ALD: Alcohol-related liver disease
ALT: Alanine aminotransferase
aPTT: Activated partial thromboplastin time
AST: Aspartate aminotransferase
AUC: Area under the concentration–time curve
BI: Boehringer Ingelheim
BID: Twice daily
BNP: Brain natriuretic peptide
BP: Blood pressure
cACLD: Compensated advanced chronic liver disease
CAP: Controlled attenuation parameter
CK18 M30: Caspase-cleaved cytokeratin 18
CK18 M65: Total cytokeratin 18
CK19: Cytokeratin 19
C_max_: Maximum concentration
C_pre_: Pre-dose concentration
CSPH: Clinically significant portal hypertension
CTCAE: Common Terminology Criteria for Adverse Events
CYP: Cytochrome P450
D: Day
DMP: Data Management Plan
ECG: Electrocardiogram
eGFR: Estimated glomerular filtration rate
ELF: Enhanced liver fibrosis
EoS: End of study
EoT: End of treatment
HBV: Hepatitis B virus
HCV: Hepatitis C virus
HOMA-IR: Homeostatic Model Assessment for Insulin Resistance
HR: Heart rate
hs-CRP: High-sensitivity C-reactive protein
HVPG: Hepatic venous pressure gradient
IED: Independent ethics committee
IRB: Institutional review board
IRT: Interactive Response Technology
LSM: Liver stiffness measurement
MELD: Model for End-Stage Liver Disease
MELD-Na: Model for End-Stage Liver Disease-sodium
MRD: Multiple rising dose
NASH: Non-alcoholic steatohepatitis
NO: Nitric oxide
NSBB: Non-selective beta-blocker
PG: Pressure gradient
PK: Pharmacokinetic
PRO: Patient-reported outcome
Pro-C3: Amino-terminal propeptide of type III collagen
Pro-C6: Propeptide of type VI collagen
PT/INR: Prothrombin time/international internalised ratio
QD: Once daily
SAE: Serious adverse event
sGC: Soluble guanylyl cyclase
SSM: Spleen stiffness measurement
T2DM: Type 2 diabetes mellitus
TE: Transient elastography
TIMP1: Tissue inhibitor of metalloproteinase 1
*t*_max_: Time to maximum concentration
W: Week
